# Evaluation of two *Plasmodium vivax* sexual stage antigens as transmission-blocking vaccine candidates

**DOI:** 10.1186/s13071-021-04909-w

**Published:** 2021-08-16

**Authors:** Yongzhe Zhang, Fei Liu, Yan Zhao, Fan Yang, Jie Bai, Xitong Jia, Wanlapa Roobsoong, Jetsumon Sattabongkot, Liwang Cui, Yaming Cao, Enjie Luo, Meilian Wang

**Affiliations:** 1 Department of Pathogen Biology, College of Basic Medical Sciences, China Medical University, Shenyang, 110122 Liaoning China; 2 Department of Nephrology, Shengjing Hospital of China Medical University, Shenyang, 110004 Liaoning China; 3 Department of Immunology, College of Basic Medical Sciences, China Medical University, Shenyang, 110122 Liaoning China; 4 Mahidol Vivax Research Unit, Faculty of Tropical Medicine, Mahidol University, Bangkok, Thailand; 5 Department of Internal Medicine, Morsani College of Medicine, University of South Florida, 3720 Spectrum Boulevard, Suite 304, Tampa, FL 33612-9415 USA

**Keywords:** *Plasmodium vivax*, Transmission-blocking vaccine, Yeast expression, Direct membrane feeding assay

## Abstract

**Background:**

*Plasmodium vivax* transmission-blocking vaccines (TBVs) are receiving increasing attention. Based on excellent transmission-blocking activities of the PbPH (PBANKA_0417200) and PbSOP26 (PBANKA_1457700) antigens in *Plasmodium berghei*, their orthologs in *P. vivax*, PVX_098655 (PvPH) and PVX_101120 (PvSOP26), were selected for the evaluation of their potential as TBVs.

**Methods:**

Fragments of PvPH (amino acids 22–304) and PvSOP26 (amino acids 30–272) were expressed in the yeast expression system. The recombinant proteins were used to immunize mice to obtain antisera. The transmission-reducing activities of these antisera were evaluated using the direct membrane feeding assay (DMFA) using *Anopheles dirus* mosquitoes and *P. vivax* clinical isolates.

**Results:**

The recombinant proteins PvPH and PvSOP26 induced robust antibody responses in mice. The DMFA showed that the anti-PvSOP26 sera significantly reduced oocyst densities by 92.0 and 84.1% in two parasite isolates, respectively, whereas the anti-PvPH sera did not show evident transmission-reducing activity. The variation in the DMFA results was unlikely due to the genetic polymorphisms of the two genes since their respective sequences were identical in the clinical *P. vivax* isolates.

**Conclusion:**

PvSOP26 could be a promising TBV candidate for *P. vivax*, which warrants further evaluation.

**Graphical Abstract:**

**Figure:**
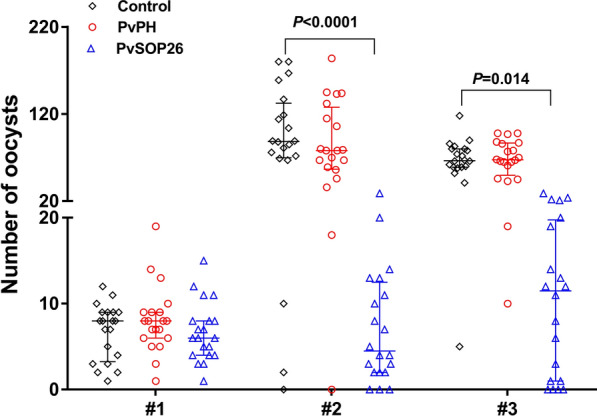

**Supplementary Information:**

The online version contains supplementary material available at 10.1186/s13071-021-04909-w.

## Background

Malaria, transmitted through the bites of infected female* Anopheles* mosquitoes, is a widespread infectious disease in the tropics. In 2019, there were 229 million reported malaria cases in the world, resulting in more than 409,000 deaths [1]. *Plasmodium vivax* is one of four human malaria parasites, with malaria caused by this parasite characterized by the recurrence of clinical symptoms every 48 h (tertian). Although less virulent than *Plasmodium falciparum*, *P. vivax* still poses a significant burden on the economy and public health in Asia and the Americas [2]. Some malaria-infected people are asymptomatic but still infectious to mosquitoes and possibly serve as an important reservoir that contributes to the sustained transmission of malaria [3]. In addition, large proportions of *P. vivax* infections are due to relapse from activation of the dormant hypnozoites in the liver [4, 5]. Vaccines hold great promise for the prevention, control and elimination of malaria.

The malaria parasite life-cycle is complex and, therefore, multi-stage vaccines targeting antigens expressed in different parasite life stages are advocated. Transmission-blocking vaccines (TBVs) target the parasite’s sexual stage antigens or the mosquito’s midgut antigens, interrupting the parasite’s transmission through mosquitoes. TBVs are intended to induce herd immunity in the population without directly protecting the vaccinated individual; thus, they may also enhance the function of vaccines or drugs by preventing the spread of resistant parasites [6, 7]. An ideal TBV should inhibit multiple steps of sexual development, including gametocytogenesis, gametogenesis, fertilization and the maturation of the ookinete and its traversal of the mosquito midgut. Unfortunately, despite decades of research efforts, only a few TBV candidates have shown clear transmission-blocking (TB) activities. These TBV candidates mainly include the pre-fertilization antigens (P230, P48/45, P47 and HAP2) [8–11], the post-fertilization antigens (P25 and P28) [12] and the *Anopheles* mosquito midgut alanyl aminopeptidase N (AnAPN1) [13, 14]. The histidine-tagged Pvs25 expressed in yeast, Pvs25H, is the first *P. vivax* TBV to enter a phase 1 clinical trial [15]. The Pvs25H protein with the adjuvant alhydrogel elicited antibodies that showed TB activity in the direct membrane feeding assay (DMFA). However, the second trial of Pvs25H with Montanide ISA 51, an oil-based immune adjuvant, was halted due to unexpected reactogenicity of the vaccine candidate in volunteers [16]. The other two promising candidates, Pvs28 and AnAPN1, are still in pre-clinical trials [17, 18]. Thus, there is a pressing need to identify additional TBV candidates for *P. vivax*.

In recent years, the genomic, transcriptomic and proteomic data available for malaria parasites provide opportunities for systematic genome-wide exploration of TBV antigens [19]. In previous studies, data mining of the PlasmoDB database led to the identification of two potential TBV candidate antigens, PbPH and PbSOP26, in the rodent parasite *Plasmodium berghei* [20, 21]. PbPH contains a pleckstrin homology (PH) domain and is expressed on the surface of gametes, zygotes and ookinetes. Functional studies revealed that knockout of the *pbph* gene affected the formation of gametocytes, gametes, ookinetes and oocysts [21]. The * PSOP26* gene encodes a putative secreted ookinete protein (PSOP) and is one of the most highly expressed genes in ookinetes [20]. Both antigens demonstrated satisfactory TB activities* in vivo* in the rodent malaria system [20, 21]. In this study, the TB potentials of the orthologs of these two antigens in *P. vivax*, PvPH and PvSOP26, were further explored. Using clinical *P. vivax* isolates from malaria patients, the DMFA demonstrated that PvSOP26 antisera significantly reduced the mosquito infection intensity for two of the three isolates.

## Methods

### Antigen selection and expression in yeast

Based on the excellent transmission-reducing (TR) activities of PbPH (PBANKA_0417200) and PbSOP26 (PBANKA_1457700) in *P. berghei* [20, 21], their orthologs in *P. vivax*, PVX_098655 (PvPH) and PVX_101120 (PvSOP26), were selected for evaluation. The sequences corresponding to amino acids (aa) 22–304 of PvPH and aa 30–272 of PvSOP26 of the Sal-I strain were synthesized and subjected to codon optimization for expression in the yeast *Pichia pastoris* (GenScript Biotech Corp., Hong Kong, China). The N-terminus of the target antigen was fused with a 6xHis-tag. There are seven and 11 putative N-glycosylation sites in PvPH and PvSOP26, respectively, and these were not mutated; they were cloned into the pPIC9K vector (Invitrogen, Thermo Fisher Scientific, Waltham, MA, USA), which was then used to transform the *Plasmodium pastoris* GS115 strain [22]. The yeast strains expressing the two recombinant proteins (rPvPH and rPvSOP26) were cultured in 1 l of buffered minimal medium and induced by methanol. After lysis of the yeast cells with an ATS high-pressure homogenizer (ATS Engineering Gmbh, Dresden, Germany), the recombinant proteins were purified using Ni–NTA columns, and the purity of the recombinant proteins was estimated by sodium dodecyl sulfate–polyacrylamide gel electrophoresis [23]. A glutathione *S*-transferase (GST) protein was used as a negative control, as described previously [24].

### Generation of anti-rPvPH and anti-rPvSOP26 sera

To generate antisera against the two recombinant proteins, we injected female BALB/c mice (*n* = 10 in each group) subcutaneously with the purified rPvPH, rPvSOP26 or GST control protein (50 μg each) emulsified in the complete Freund’s adjuvant (Sigma-Aldrich, St Louis, MO, USA). The mice were then given two more booster immunizations with the same recombinant proteins (25 μg/mouse) emulsified in incomplete Freund’s adjuvant (Sigma-Aldrich) at 2-week intervals. Finally, 2 weeks after the third immunization, the antisera in each group of mice were collected* via* cardiac puncture and pooled.

### Western blot

The recombinant proteins rPvPH and rPvSOP26 were separated in 12% SDS–PAGE gels under reduced conditions and electro-transferred onto a 0.22-μm polyvinylidene fluoride membrane (Bio-Rad, Hercules, CA, USA). After blocking with 5% non-fat milk in Tris-buffered saline with 0.1% Tween 20 for 2 h, the blots were probed with the pooled mouse antisera (1:200 dilution) against rPvPH or rPvSOP26 as the primary antibodies and horseradish peroxidase (HRP)-conjugated goat anti-mouse immunoglobulin G (IgG) antibody (1:10,000) as the secondary antibodies. A Western blot kit (Thermo Fisher Scientific) was used to visualize the protein bands [25].

### Enzyme-linked immunosorbent assay

An enzyme-linked immunosorbent assay (ELISA) was performed to determine the antibody titers of mouse immune sera. Microtiter plates were coated with the purified rPvPH or rPvSOP26 (5 μg/ml) at 4 °C for > 8 h. The plates were first blocked with 1% bovine serum albumin (BSA) for 2 h at 37 °C, then incubated with the antisera from mice immunized with rPvPH and rPvSOP26, respectively, at 37 °C for 2 h. These pooled antisera from the control and immunization groups were twofold serially diluted in 1% BSA in phosphate-buffered saline (PBS) from 1:200 to 1:512000. After two washes with PBS, 100 μl HRP-conjugated goat anti-mouse IgG antibodies (Invitrogen Thermo Fisher Scientific; 1:5,000) was added to each well and incubated for 2 h. After five washes with PBS, tetramethylbenzidine was added to each well, and the plate was kept in the dark for 10 min. The reaction was stopped by adding 2 mM H_2_SO_4_. An ELISA plate reader was used to measure the absorbance at 490 nm [26]. The endpoint titers were determined as the highest antiserum dilution with an optical reading greater than the average reading from a control serum (anti-GST) plus three standard deviations (SD) as the cut-off value [26].

### *Plasmodium vivax* clinical samples

The human subject protocol for this study was approved by the Ethics Committee of the Faculty of Tropical Medicine, Mahidol University, Bangkok, Thailand (MUTM 2018-016). Patients with *P. vivax* malaria who were symptomatic for clinical malaria, slide-positive for *P. vivax* infection, aged ≥ 18 years and not pregnant were considered eligible for inclusion in this study. Three *P. vivax* patients were enrolled after signing the informed consent form. Before the initiation of antimalarial treatment, 5–10 ml venous blood was collected into heparinized tubes and used to make blood smears and for the DMFA [27].

### Indirect immunofluorescence assay

The expression and the location of PvPH were studied using the indirect immunofluorescence assay (IFA). The erythrocytes from the *P. vivax* patients were mixed with 47% Nycodenz-supplemented RPMI 1640 medium and centrifuged at 500* g* for 25 min to obtain the parasite-infected erythrocytes at the gray interface. The latter were used to make thin smears, which were fixed with 4% paraformaldehyde for 30 min at 37 °C. Skimmed milk (5%) in PBS was used to block the slides for 30 min. After three washes with PBS, the slides were incubated with mouse antisera (1:500) against rPvPH, rPvSOP26 or the GST control for 1 h at room temperature. After three washes with cold PBS, the slides were incubated with fluorescein isothiocyanate (FITC)-conjugated goat anti-mouse antibodies (1:500; Invitrogen Thermo Fisher Scientific) for 1 h, and 1 μg/ml 4′6-diamidino-2-phenylindole (DAPI; Invitrogen Thermo Fisher Scientific) for 30 min. After an additional wash with cold PBS, the slides were mounted with the ProLong®Gold Antifade Reagent kit (Invitrogen Thermo Fisher Scientific). Fluorescence images were observed with an Olympus BX53 microscope (Olympus Cop., Tokyo, Japan) and processed using Adobe Photoshop (Adobe Inc., San Jose, CA, USA) [25, 28].

### Quantification of TB activity

Antisera from mice immunized with rPvPH, rPvSOP26 or the GST control protein were diluted with heat-inactivated AB+ serum obtained from healthy donors in Thailand (1:1, v/v). Erythrocytes of *P. vivax* patients were mixed with the diluted sera (1:1, v/v) and incubated at 37 °C for 15 min. Each reconstituted infected blood sample was then introduced to a glass feeder and kept at 37 °C. One hundred starved mosquitoes were allowed to feed on the blood mixture for 30 min at 37 °C through the membrane feeder. After several hours, fully engorged mosquitoes were identified, isolated, and kept on a 10% sucrose solution in cotton balls at 20 °C at 80% relative humidity for 1 week. Twenty mosquitoes from each group were randomly dissected on day 7 after blood-feeding. Mosquito midguts were stained with 5% mercurochrome, and oocysts were counted [10, 29]. The infection prevalence, which is the proportion of oocyst-positive infected mosquitoes, was used to determine TB activity. The intensity of infection, i.e. the number of oocysts per mosquito midgut, was used to determine TR activity.

### Analysis of genetic polymorphisms

Genetic polymorphisms of the PvPH and PvSOP26 genes were determined for the parasite isolates used in the DMFA. DNA was extracted from dried filter-paper blood spots using a QIAamp DNA Blood Mini kit (Qiagen, Hilden, Germany). DNA fragments encoding rPvPH (22–304 aa) and rPvSOP26 (30–272 aa) were amplified by PCR [24]. The primers were designed based on the *P. vivax* Sal-1 (PVX_083235) sequence: PvPH-F (GTCCCAATTAGAATCTGTTT) and PvPH-R (GTTCCTTCTGTTGGGTGTTT); PvSOP26-F (ACCTTGTAGCCTCTACACTT) and PvPH-R (AAATTTGTTGAAAAAATTAT). All amplified DNA products were purified with a QIAquick Gel Extraction kit (Qiagen) and sequenced using the ABI Prism® BigDye™ cycle sequencing kit (Applied Biosystems, Thermo Fisher Scientific) as previously described [30]. Alignment of nucleotide sequences was done using BioEdit software.

### Statistical analyses

Statistical analyses were performed using SPSS version 22.0 software (SPSS IBM Corp., Armonk, NY, USA). The Student t-test was used for comparison of antibody titers between the control and immunization groups. The intensity of infection was compared using the Mann–Whitney U-test. The infection prevalence was compared using Fisher’s exact test. *P* values of < 0.05 were considered to be statistically significant.

## Results

### Identification, expression and purification of PvPH and PvSOP26

**Fig. 1:**
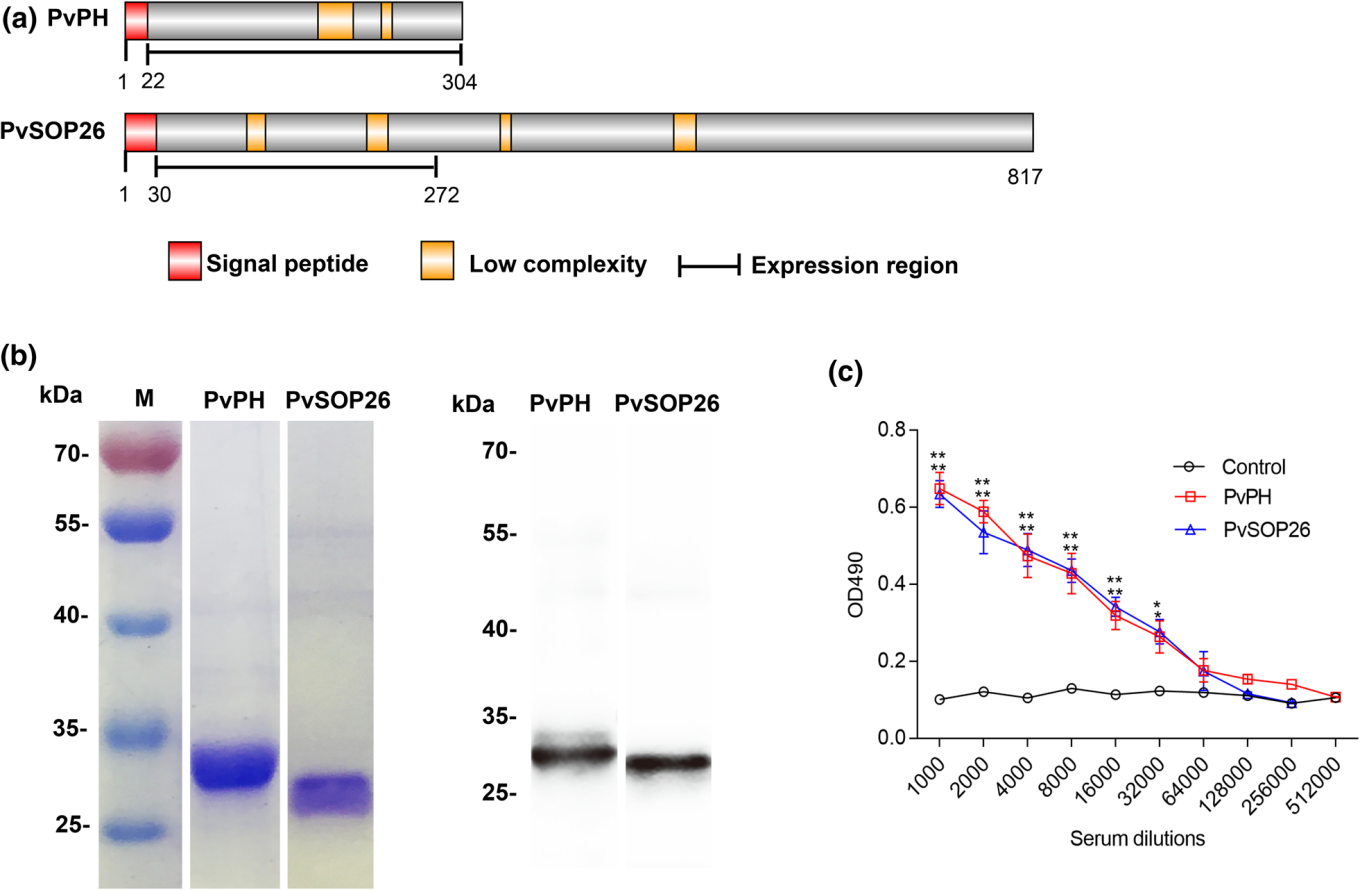
Domain organization, expression and immunization of PvPH and PvSOP26. **a** Schematics of PvPH and PvSOP26 showing domain organization and fragments expressed in yeast. Signal peptide, transmembrane regions, low complexity regions and the expressed segment are illustrated. **b** Sodium dodecyl sulfate–polyacrylamide gel electrophoresis (SDS-PAGE) and western blot analysis of recombinant PvPH and PvSOP26. The left and middle panels show Coomassie-stained SDS-PAGE gels of the purified recombinant proteins. The right panel shows the western blot analysis using the mouse antisera against the respective recombinant proteins.* M* Protein marker in kiloDaltons. **c** Antibody titers against the recombinant proteins determined by enzyme-linked immunosorbent assay. Pooled serum samples were tested at twofold serial dilutions. Results are representative of three independent experiments. Bars (error bars) indicate the mean (±  standard error of the mean [SEM]) for three mice per group. **P < 0.01 (Student’s t-test).1 :32000 control* vs* PvPH:* t*_(2)_ = − 7.64, *P* = 0.016; 1:32000 control* vs* PvSOP26: *t*_(2)_ = − 10.733, *P* = 0.016

A search of PlasmoDB for the orthologs of PbPH and PbSOP26 identified the PVX_098655 and PVX_101120 genes in *P. vivax*, designated as PvPH and PvSOP26, respectively. Multiple sequence alignment revealed that these two genes are highly conserved among the *Plasmodium* species (Additional file 1: Figure S1). PvPH and PvSOP26 showed 55.6% and 27.7% identity in amino acids to their respective orthologs in *P. berghei*. A 282-aa fragment (22–304 aa) of PvPH and a 242-aa fragment (30–272 aa) of PvSOP26 were selected for expression in the yeast *P. pastoris* (Fig. 1a). Each of the recombinant proteins was expressed in 1 l of culture and purified using Ni–NTA chromatography. The yield of both recombinant proteins was approximately 1000 mg/l. SDS–PAGE analysis showed that the purified recombinant rPvPH and rPvSOP26 were approximately 32 and 29 kDa, respectively, consistent with predicted molecular weights (Fig. 1b).

### Immunization of mice with the recombinant proteins

The purified recombinant proteins were used to immunize mice to raise polyclonal antibodies. ELISA using the pooled immune sera for each antigen collected 2 weeks after the final booster showed excellent immunogenicity of the recombinant proteins, and the antibody titers for both rPvPH and rPvSOP26 reached 1:32,000 (Fig. 1c; 1:32000 control* vs* PvPH:* t*_(2)_ = − 7.64, *P* = 0.016; 1:32000 control* vs* PvSOP26: *t*_(2)_ = − 10.733, *P* = 0.016). Western blots showed that the antisera against rPvPH and rPvSOP26 recognized the respective recombinant proteins (Fig. 1b).

### PvPH expression at the *P. vivax* gametocyte stage

**Fig. 2:**
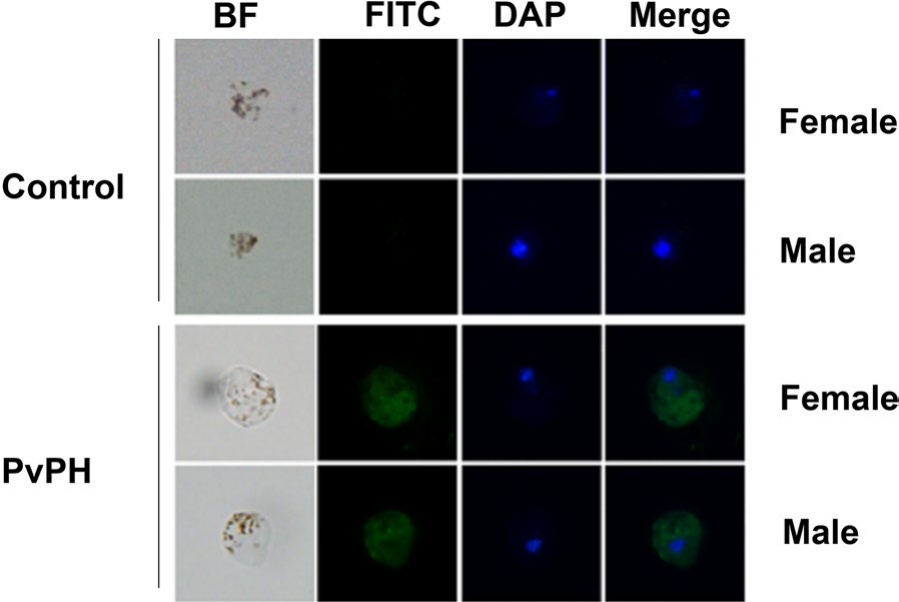
Indirect immunofluorescence assay detection of PvPH in *Plasmodium vivax* gametocytes. Gametocyte-infected erythrocytes were probed with the mouse anti-PvPH sera or the control anti-glutathione *S*-transferase (GST) sera. Fluorescein isothiocyanate (*FITC*)-conjugated goat anti-mouse immunoglobulin G (green) was used as the secondary antibodies. Nuclei were stained with 4′6-diamidino-2-phenylindole (*DAP*; blue). The images were magnified at ×1000.* BF*, bright field. Scale bar: 5 μm

An IFA was performed to determine whether PvPH was expressed at the gametocyte stage of *P. vivax*. IFA with mouse anti-rPvPH sera detected a fluorescent signal in *P. vivax* gametocytes from a clinical isolate compared to the negative control with mouse anti-GST sera (Fig. 2). This result is consistent with the expression of PvPH during gametocyte development in *P. vivax*. Due to difficulties in culturing *P. vivax* ookinetes, PvSOP26 protein expression was not examined.

### TR activity of the mouse antisera

**Fig. 3:**
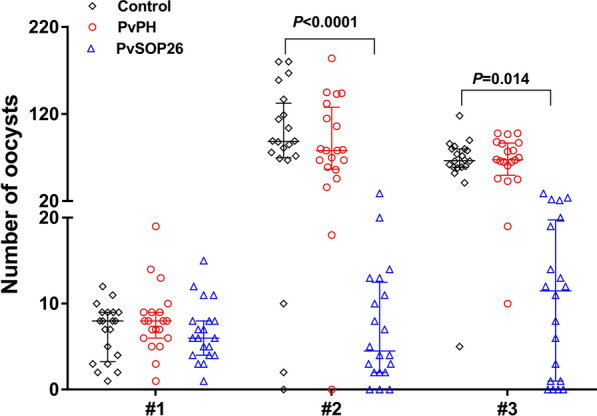
Evaluation of transmission-reducing activities of mouse antisera against PvPH, PvSOP26, and GST with *Plasmodium vivax* clinical isolates. A direct membrane feeding assay was performed for three *P. vivax* isolates (#1, #2 and #3, respectively, on the* x*-axis) with reconstituted blood consisting of mouse antisera mixed with heat-inactivated AB-positive blood type healthy human serum at 1:1. The numbers of oocysts in individual mosquito midguts are shown in the scatter dot plot. The long horizontal bar designates the median number of oocysts, while the two short horizontal bars indicate interquartile ranges in each group. * and *** indicate significant differences at *P* < 0.05 and *P* < 0.001, respectively (Mann–Whitney U-test). #2 control* vs* PvSOP26: *U*_(19)_ = 40.5, *Z* = − 4.32, *P* < 0.0001; #3 control *vs* PvSOP26: *U*_(19)_ = 13, *Z* = − 5.06, *P* = 0.014

To evaluate whether mouse anti-rPvPH and anti-rPvSOP26 sera had TR activity, a DMFA was carried out with clinical samples obtained from three *P. vivax* patients using laboratory-reared *An. dirus* mosquitoes (*P. vivax* samples #1, #2 and #3 in Table 1). While the blood from donor #1 had an infection prevalence of 100% compared with the control sera (for GST) and immune sera against PvPH and PvSOP26, the oocyst densities in all groups were low (mean 6.7–8.1 oocysts/midgut). Neither antisera showed noticeable TR activity (Table 1; Fig. 3). For the control antisera, the other two donor blood samples infected 95–100% mosquitoes and had high oocyst densities (67.8–94.4 oocysts/midgut). Compared with the control group, the anti-rPvSOP26 group showed significantly reduced oocyst density by 92.0% and 84.1%, respectively (Table 1; Fig. 3, #2 control* vs* PvSOP26: *U*_(19)_ = 40.5, *Z* = − 4.32, *P* < 0.0001; #3 control *vs* PvSOP26: *U*_(19)_ = 13, *Z* = − 5.06, *P* = 0.014). The PvSOP26 antisera also showed a modest reduction of infection prevalence by 10 and 20%, respectively (*P* = 1.0 and *P* = 0.106). In contrast, the anti-rPvPH sera showed no apparent TB or TR activity regarding infection prevalence and oocyst density (Table 1; Fig. 3).

**Table 1:** Prevalence of *Plasmodium vivax* infection and oocyst numbers in mosquitoes fed on antisera

*P. vivax* clinical samples	Group	Oocyst density, median (IQR)	Mean oocyst density (oocysts/midgut)	% inhibition of oocyst density^a^	*P-*value^b^	Infected/dssected^c^	Infection rate (%)	% inhibition of prevalance^d^	*P-*value^e^
#1	Control	8.0 (3.3–9.0)	6.8			20/20	100		
	PvPH	8.0 (6.0–9.0)	8.1	− 19.1	0.933	20/20	100	-	
	PvSOP26	6.0 (4.0–8.0)	6.7	0.7	1.000	20/20	100	-	1.000
#2	Control	88.5 (69.8–132.5)	94.4			19/20	95		
	PvPH	78.0 (56.8–127.8)	85.2	9.8	1.000	19/20	95	-	
	PvSOP26	4.5 (2.0–12.5)	7.5	92.0	0.0001***	17/20	85	10	1.000
#3	Control	66.5 (59.0–80.0)	67.8			20/20	100		
	PvPH	67.5 (49.8–86.8)	66.7	1.6	1.000	20/20	100	-	
	PvSOP26	11.5 (1.0–19.8)	10.80	84.1	0.014*	16/20	80	20	0.106

*IQR* Inter-quartile range

*P < 0.05, ***P < 0.001

^a^Percentage inhibition of oocyst density was calculated as (mean_control_ − mean_PvPH/PvPSOP26_)/mean_control_ × 100%

^b^Mean number of oocysts was statistically analyzed (Mann–Whitney *U*-test) and *P*-values of < 0.05 were considered to be statistically significant. #2 control *vs*. PvSOP26: *U*_(19)_ = 40.5, *Z* = − 4.32, *P* < 0.0001; #3 control *vs* PvSOP26: *U*_(19)_ = 13, *Z* = − 5.06, *P* = 0.014

^c^Infection prevalence was calculated by number of oocyst-infected mosquitoes per 20 mosquitoes dissected in each group (Infected/dissected)

^d^% inhibition of prevalence was calculated as % prevalence_control_ − % prevalence_PvPH/PvPSOP26_

^e^Prevalance was statistically analyzed by Fisher’s exact test. *P*-values < 0.05 were considered to be statistically significant

### Genetic polymorphisms

To determine whether the variations in TR activity may be related to genetic polymorphisms of the target antigens since the antibodies were generated against the sequences of the Sal-I strain, the DNA fragments of PvPH and PvSOP26 were sequenced in the three *P. vivax* samples. Compared to the Sal-I sequences, the PvPH gene in the three samples showed no amino acid substitution, whereas all three *P. vivax* isolates had the same substitutions, K263N, I355S and L403I. in the PvSOP26 gene.

## Discussion

Based on the excellent TB potentials of PbPH and PbSOP26 from a TBV discovery effort using the rodent malaria system [20, 21], the orthologs of these proteins were evaluated as TBV candidates in *P. vivax*. IFA with the mouse antisera confirmed PvPH expression in the gametocyte stage of *P. vivax*. When the immune sera against the recombinant PvPH and PvSOP26 were evaluated using DMFA with *P. vivax* clinical isolates, the mouse anti-PvSOP26 antisera demonstrated considerable TR activities in reducing oocyst density.

For the development of recombinant protein-based TBVs, expression of the recombinant proteins with properly folded conformational epitopes can be critical for inducing antibodies with TR activities [10, 31]. The yeast protein expression system has been widely used to produce many human vaccines [32, 33]. Compared to the prokaryotic expression system, the yeast system has the advantage of higher biomass expression and secretion yields. It also offers better protein-folding, disulfide-bond formation and similar post-translational modifications as in mammals [10, 34]. For malaria vaccine development, the circumsporozoite protein, merozoite surface protein 1 and apical membrane antigen 1 expressed in yeast produces effective and protective antibodies in mice [35–39]. The yeast expression system has also been used to express TBV candidates such as Pvs25and Pfs25 with multiple disulfide bonds [40, 41]. In the present study, the *P. pastoris* expression system was used to express PvPH and PvSOP26 with a yield of  approximately1 g/l of yeast culture, and the recombinant proteins showed excellent immunogenicity. The resulting antibodies were able to recognize the native proteins expressed in *P. vivax* gametocytes.

Safe and effective adjuvants play an equally important role in vaccine research. One limitation of this study is the use of Freund’s adjuvants for boosting immune responses, which are not suitable for use in humans. Several adjuvants have been tested in human clinical trials of TBVs, including Montanide ISA 51 and EPA/Alhydrogel [15, 16, 42]. Glucopyranosyl lipid adjuvant-stable emulsion has been demonstrated to induce high levels of antibodies against the Pfs48/45-GLURP chimera in experimental models [43], and it also had a good safety profile in human clinical trials of malaria vaccines [44]. Future studies should evaluate promising TBV candidates with adjuvants suitable for human use.

PSOP26 is predicted to be a secreted protein in ookinetes [45]. It is a highly expressed protein in *P. berghei* ookinetes, as its transcript ranked in the 99th percentile in the ookinete transcriptome. In mice infected by *P. berghei*, immunization with the recombinant PbSOP26 showed significant TB and TR activities in direct feeding assays (DFAs) [20]. In this study, the expressed fragment of PvSOP26 covered the entire domain of the PbSOP26 used for TBV analysis [20]. The immune sera against PvSOP26 were evaluated using DMFA with three clinical *P. vivax* isolates. When the *P. vivax* clinical isolates resulted in high oocyst density (> 50 oocysts/midgut) in infected mosquitoes in the control group (as in donor #2 and #3), the anti-PvSOP26 sera significantly reduced the oocyst density by > 84%. It is not clear why the anti-PvSOP26 antisera did not show TR activities with parasites from donor #1 when the infection intensity in mosquitoes was low. One possible explanation is variations in the DMFA; additional patient isolates needed to be tested to provide further evidence of the TR activity of PvSOP26. While the results of this study suggest that genetic polymorphisms might not be responsible for the variation in the DMFA result among the clinical isolates, further analysis is needed to understand the genetic diversity of these genes. In this regard, the orthologs of these genes in *P. falciparum* showed limited genetic diversity from high-throughput genome sequencing projects (https://plasmodb.org/).

The PH domain is predicted in most *Plasmodium* PH orthologs [21]. The PH domain can bind phosphatidylinositol in biological membranes, thus recruiting or targeting the proteins to the membrane fraction [46]. Mice immunized with recombinant PbPH protein reduced both the infection prevalence and oocyst density in the DFAs [21]. Despite the high level of homology between the PvPH and PbPH, antibodies generated against the PvPH fragment did not show any TR activities in the DMFA with *P. vivax* clinical isolates. Several reasons may account for the lack of TR activities of the anti-PvPH antibodies. Although the anti-PvPH antisera could detect the antigen expression in *P. vivax* gametocytes, the antibodies may recognize those epitopes that are not critical for fertilization and sexual development. Also, although the rodent model offers the convenience for antigen discovery where a DFA can be performed [47], the ortholog may not have identical functions in *P. vivax*. The use of transgenic *P. berghei* expressing *P. vivax* full-length target genes may offer a better prediction for the TBV potential [48, 49].

## Conclusion

We evaluated two sexual stage antigens, PvPH and PvSOP26, as TBV candidates using *P. vivax* clinical isolates. PvSOP26 showed prominent TR activities in reducing oocyst density in two of the three mosquito feeding assays.

## Supplementary Information

**Additional file 1**: **Figure S1**. Alignment analysis of protein sequences of PvPH and PvSOP26 in *Plasmodium* spp.: *P. vivax* (Pv), *P. knowlesi* (Pk), *P. falciparum* (Pf) and *P. berghei* (Pb).

## Data Availability

The datasets supporting the conclusions of this article are included within the article and its additional file.

## Abbreviations

AnAPN1: *Anopheles* Mosquito midgut alanyl aminopeptidase N
BSA: Bovine serum albumin
DAPI: 4′6-Diamidino-2-phenylindole
DMFA: Direct membrane feeding assay
ELISA: Enzyme-linked immunosorbent assay
GST: Glutathione *S*-transferase
IFA: Indirect immunofluorescence assay
PBS: Phosphate-buffered saline
PH: Pleckstrin homology
PSOP: Putative secreted ookinete protein
SDS-PAGE: Sodium dodecyl sulfate–polyacrylamide gel electrophoresis
TB: Transmission-blocking
TBV: Transmission-blocking vaccine
TR: Transmission-reducing
