# Supplementary material for: Evaluation of two Plasmodium vivax sexual stage antigens as transmission-blocking vaccine candidates
Source: Parasit Vectors. 2021 Aug 16;14:407. doi: 10.1186/s13071-021-04909-w (PMC8366161; doi:10.1186/s13071-021-04909-w)
Supplement: Supplementary file 1 — Additional file 1: Figure S1. Alignment analysis of protein sequences of PvPH and PvSOP26 in Plasmodium spp.: P. vivax (Pv), P. knowlesi (Pk), P. falciparum (Pf) and P. berghei (Pb). [file 13071_2021_4909_MOESM1_ESM.docx]

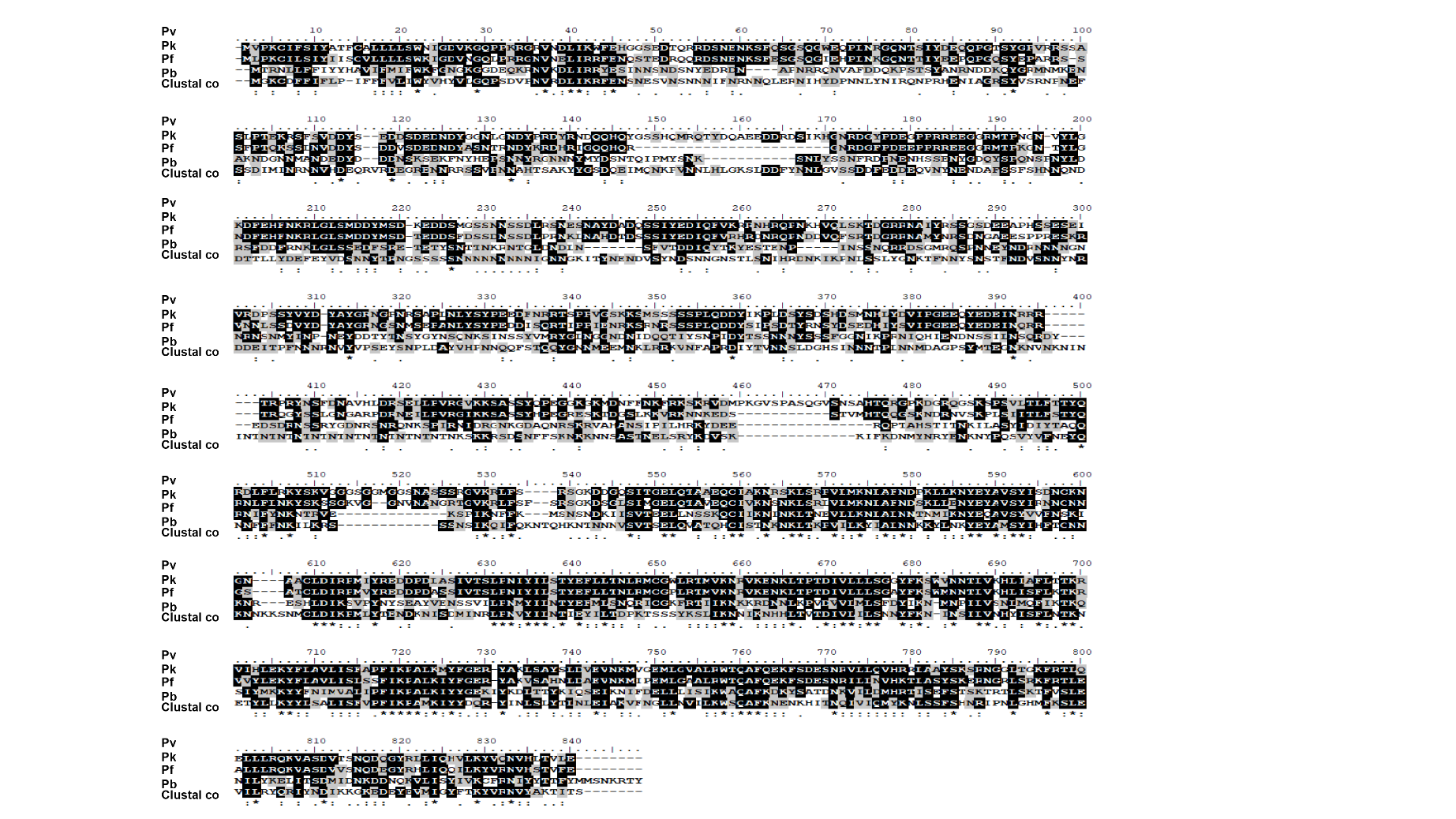

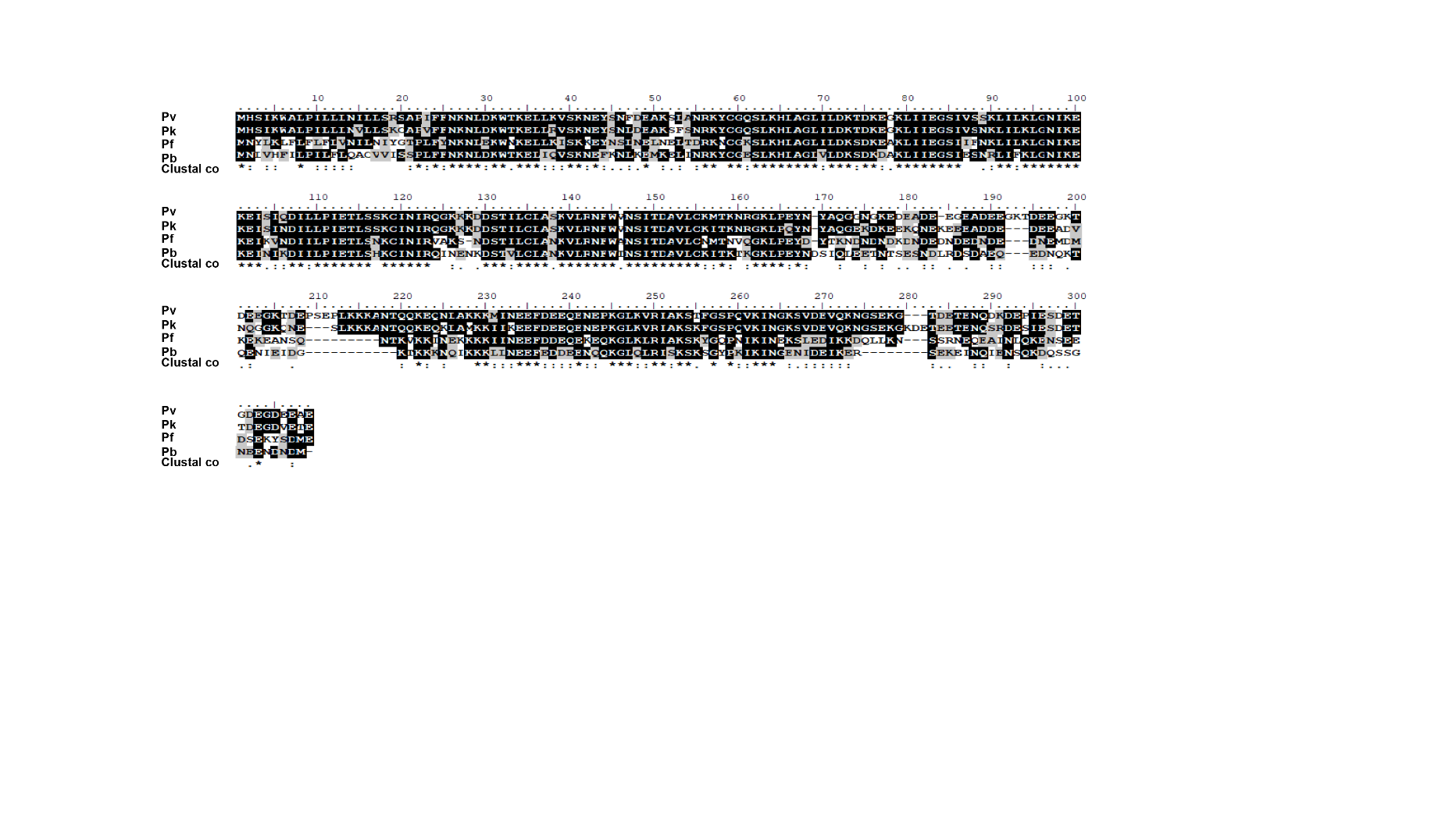


**PvSOP26**

**PvPH**

**Additional file 1: Figure S1.** Alignment analysis of protein sequences of PvPH and PvSOP26 in *Plasmodium* spp.: *P. vivax* (Pv), *P. knowlesi* (Pk), *P. falciparum* (Pf) and *P. berghei* (Pb)*.*
